# Genomic characterization of the rotavirus G3P[8] strain in vaccinated children, reveals possible reassortment events between human and animal strains in Manhiça District, Mozambique

**DOI:** 10.3389/fmicb.2023.1193094

**Published:** 2023-06-05

**Authors:** Filomena Manjate, Eva D. João, Peter Mwangi, Percina Chirinda, Milton Mogotsi, Augusto Messa, Marcelino Garrine, Delfino Vubil, Nélio Nobela, Tacilta Nhampossa, Sozinho Acácio, Jacqueline E. Tate, Umesh Parashar, Goitom Weldegebriel, Jason M. Mwenda, Pedro L. Alonso, Celso Cunha, Martin Nyaga, Inácio Mandomando

**Affiliations:** ^1^Centro de Investigação em Saúde de Manhiça (CISM), Maputo, Mozambique; ^2^Global Health and Tropical Medicine (GHTM), Instituto de Higiene e Medicina Tropical (IHMT), Universidade Nova de Lisboa (UNL), Lisbon, Portugal; ^3^Next Generation Sequencing Unit and Division of Virology, Faculty of Health Sciences, University of the Free State, Bloemfontein, South Africa; ^4^Instituto Nacional de Saúde, Ministério da Saúde, Marracuene, Mozambique; ^5^Centers for Disease Control and Prevention (CDC), Atlanta, GA, United States; ^6^African Rotavirus Surveillance Network, Immunization, Vaccines, and Development Program, Regional Office for Africa, World Health Organization, Brazzaville, Democratic Republic of Congo; ^7^ISGlobal, Hospital Clínic, Universitat de Barcelona, Barcelona, Spain

**Keywords:** children <5 years, moderate-to-severe diarrhea, rotavirus group A, human G3P[8] strains, whole-genome sequencing, reassortments

## Abstract

Mozambique introduced the rotavirus vaccine (Rotarix®; GlaxoSmithKline Biologicals, Rixensart, Belgium) in 2015, and since then, the Centro de Investigação em Saúde de Manhiça has been monitoring its impact on rotavirus-associated diarrhea and the trend of circulating strains, where G3P[8] was reported as the predominant strain after the vaccine introduction. Genotype G3 is among the most commonly detected Rotavirus strains in humans and animals, and herein, we report on the whole genome constellation of G3P[8] detected in two children (aged 18 months old) hospitalized with moderate-to-severe diarrhea at the Manhiça District Hospital. The two strains had a typical Wa-like genome constellation (I1-R1-C1-M1-A1-N1-T1-E1-H1) and shared 100% nucleotide (nt) and amino acid (aa) identities in 10 gene segments, except for VP6. Phylogenetic analysis demonstrated that genome segments encoding VP7, VP6, VP1, NSP3, and NSP4 of the two strains clustered most closely with porcine, bovine, and equine strains with identities ranging from 86.9–99.9% nt and 97.2–100% aa. Moreover, they consistently formed distinct clusters with some G1P[8], G3P[8], G9P[8], G12P[6], and G12P[8] strains circulating from 2012 to 2019 in Africa (Mozambique, Kenya, Rwanda, and Malawi) and Asia (Japan, China, and India) in genome segments encoding six proteins (VP2, VP3, NSP1-NSP2, NSP5/6). The identification of segments exhibiting the closest relationships with animal strains shows significant diversity of rotavirus and suggests the possible occurrence of reassortment events between human and animal strains. This demonstrates the importance of applying next-generation sequencing to monitor and understand the evolutionary changes of strains and evaluate the impact of vaccines on strain diversity.

## 1. Introduction

Rotavirus group A (RVA) remains the important viral etiology that causes diarrhea in children under 5 years of age (Crawford et al., 2017), having caused an estimated 128,500 deaths in 2016 (Troeger et al., 2018). The highest mortality (82% of all deaths, *N* = 104,733) was recorded in Asia and sub-Saharan Africa (Troeger et al., 2018). RVA was found to be the leading pathogen associated with moderate-to-severe diarrhea in African (Mozambican, Gambian, Kenyan, and Malian) and Asian (Bangladesh, Indian, and Pakistan) children, with the highest attributable fraction reported in Mozambican infants before the vaccine introduction (Kotloff et al., 2013). After the introduction of the RVA vaccine into the Mozambican immunization program in September 2015, there has been a decrease in RVA prevalence among children under 5 years of age, from 22.9% before the vaccine introduction to 11.5% after the vaccine introduction (Manjate et al., 2022).

Rotavirus group A is a virus with a double-stranded ribonucleic acid (dsRNA) genome composed of 11 segments enclosed in a capsid that includes three proteins that form the outer, intermediate, and inner layers (Desselberger, 2014). The virus segments encode six structural proteins (VP1-VP4, VP6, and VP7) and five or six non-structural proteins (NSP1-NSP5/NSP6) (Desselberger, 2014). RVA strain classification has been based mainly on the binominal system, using the two outer capsid proteins, VP7 protein encoded by genome segment nine (glycoprotein, designated as G-type) and VP4 protein encoded by genome segment four (protease-sensitive, designated as P-type), which stimulate the production of neutralizing antibodies (Iturriza-Gómara et al., 2004; Matthijnssens et al., 2008). However, it is preferred to use the extended RVA genotype classification system, which takes into account all 11 RVA genes (VP7-VP4-VP6-VP1-VP2-VP3-NSP1-NSP2-NSP3-NSP4-NSP5) and assigns each genotype diversity with the notation Gx-P[x]-Ix-Rx-Cx-Mx-Ax-Nx-Tx-Ex-Hx (x = an Arabic number that starts in 1, identifying the genotype diversity) (Matthijnssens et al., 2008, 2011).

Based on the whole genome sequencing of the 11 segments, RVA is further classified into three genogroups. The Wa-like genogroup of porcine origin, comprising mainly G1P[8], G3P[8], G4P[8], G9P[8], and G12P[8] genotype combinations, with genotype one backbone constellation (I1-R1-C1-M1-A1-N1-T1-E1-H1) (Heiman et al., 2008; Matthijnssens et al., 2008; Uprety et al., 2021); the DS-1-like genogroup, of bovine origin and found worldwide in human circulation, comprising genotype two backbone constellation (I2-R2-C2-M2-A2-N2-T2-E2-H2) (Matthijnssens et al., 2008; Uprety et al., 2021); notably, atypical combinations of G1P[8], G3P[8], and G9P[8] bearing a genotype two backbone constellation have been documented (Fukuda et al., 2020; Mwangi et al., 2020; Mhango et al., 2022). Finally, the third and less common genogroup is the AU-1-like genogroup which comprises the G3P[9] genotype combination with a genotype three backbone constellation (I3-R3-C3-M3-A3-N3-T3-E3-H3) (Nakagomi et al., 1990; Matthijnssens and Van Ranst, 2012). As of 02 March 2023, the rotavirus classification working group has identified 42G, 58P, 32I, 28R, 24C, 23M, 39A, 28N, 28T, 32E and 28H genotypes, based on the characterization of all the 11 gene segment of rotavirus.^1^ The RVA genome is prone to evolution, leading to frequent mutations and the emergence of new strains that can efficiently spread among both humans and animals (Cook, 2004; Jain et al., 2014). The evolution and diversity of RVA strains are a result of various events that may occur in the genome segments, including point mutations, genetic rearrangements through transmission between species, and reassortment events (Ghosh and Kobayashi, 2011; Joshi et al., 2019).

Moreover, the conventional genotyping system that only focuses on VP7 and VP4 gene segments may not effectively detect these mutations, which may occur in other genes (Nakagomi et al., 1990). Therefore, data on the complete genome segments of RVA can reveal these changes and provide a deeper understanding of the strains’ origin and diversity (Nakagomi et al., 1990). Following the introduction of RVA vaccines, the rapid spread of G3 rotavirus strains has been documented (Cowley et al., 2016; Dóró et al., 2016; Guerra et al., 2016; Kang and Cai, 2018; Pietsch and Liebert, 2018; Esposito et al., 2019; Katz et al., 2019; Mwanga et al., 2020; Gutierrez et al., 2021; Martinez-Gutierrez et al., 2021; Varghese et al., 2021). G3 strains have been described as commonly found in various animal hosts (cats, dogs, rabbits, goats, lambs, pigs, horses, cows, mice, birds, and monkeys) and humans (Martínez-Laso et al., 2009). The rate of G3 detection has been increasing, especially in countries using Rotarix (Rotarix®-GlaxoSmithKline Biologicals, Rixensart, Belgium) vaccine in their immunization programs, such as Australia (Roczo-Farkas et al., 2018), China, India, Malawi, Kenya (Mwanga et al., 2020; Mhango et al., 2022), and Mozambique (João et al., 2020; Manjate et al., 2022).

Many countries from Asia (Pakistan, Indonesia, Japan, and Thailand), North and South America (Colombia, Brazil, and the Dominican Republic), Australia, and Europe (Germany, Spain, and Hungary) have reported the predominance of equine-like G3 strains bearing P[8] (Cowley et al., 2016; Dóró et al., 2016; Pietsch and Liebert, 2018; Esposito et al., 2019; Katz et al., 2019; Gutierrez et al., 2021; Martinez-Gutierrez et al., 2021). These G3P[8] strains are described as being new equine-like variants, presenting a DS-1-like genotype constellation (G3P[8]-I2-R2-C2-M2-A2-N2-T2-E2-H2) (Cowley et al., 2016; Dóró et al., 2016; Guerra et al., 2016; Pietsch and Liebert, 2018; Esposito et al., 2019; Katz et al., 2019; Gutierrez et al., 2021; Martinez-Gutierrez et al., 2021), although the majority of globally circulating G3P[8] (Matthijnssens et al., 2011) strains possess a Wa-like constellation, I1-R1-C1-M1-A1-N1-T1-E1-H1 (Perkins et al., 2017). Data on the characterization of G3 strains circulating in African countries are scarce and need to be elucidated. In Malawi, G3 strains were highly detected between 1997–1999 before vaccine introduction and re-emerged after nearly two decades (2017), becoming predominant (Mhango et al., 2022). In Mozambique, we observed a change in the pattern of rotavirus circulating strains, where the combinations G1P[8], G2P[4], G12P6, and G12P[8] were the most frequent before the vaccine introduction, and there was an emergence of G3P[4] and G3P[8] after vaccine introduction (Langa et al., 2016; João et al., 2018, 2020; Manjate et al., 2022). In this study, we report on the whole genome constellation of the emerging G3P[8] detected in children hospitalized in the Manhiça District Hospital (in southern rural Mozambique) with moderate-to-severe diarrhea.

## 2. Methodology

### 2.1. Site description and sample source

This study is part of the ongoing surveillance of diarrheal diseases established at the Centro de Investigação em Saúde de Manhiça (CISM) to assess over time epidemiological trends and the etiology of diarrhea, as well as monitor the impact of rotavirus vaccination. This surveillance has been established in the Manhiça District alongside other morbidity and demographic platforms, as previously described (Sacoor et al., 2013; Nhacolo et al., 2021). In this study, we sequenced positive RVA samples of children <5 years of age that had been genotyped by conventional reverse transcription polymerase chain reaction (RT-PCR) to identify VP7 and VP4 gene segments (G/P genotypes), according to previously described protocols (Gouvea et al., 1990; Gentsch et al., 1992; Mphahlele et al., 1999; Iturriza-Gómara et al., 2000; Iturriza Gómara et al., 2004; Aladin et al., 2010). G3P[8] were selected for sequencing because of their emergence and higher frequency in surveillance. Two G3P[8] strains were selected for deep characterization. The strains were detected in two children aged 18 months old hospitalized at the Manhica District Hospital with moderate-to-severe diarrhea in 2021. One child had a history of fever, vomiting, lethargy, and sunken eyes, while the other child had only sunken eyes. Both children were female, had four diarrhea episodes that lasted 24 h and received intravenous rehydration. They had been fully vaccinated with two doses of the Rotarix© vaccine.

### 2.2. dsRNA extraction and purification

Virus dsRNA was extracted from the stool samples using the TRIzol™ reagent, as previously described (Potgieter et al., 2009), albeit with minor modifications. Briefly, approximately 500 mg of fresh stool was added to 1 mL of TRIzol™ reagent (Invitrogen, Carlsbad, CA) and incubated at room temperature for 5 min; and then centrifuged at 16,000 RPM for 15 min at 4°C (same centrifugation conditions for the next steps). Thereafter, 300 μL of chloroform (Sigma-Aldrich®, St. Louis, MO, United States) was added to the solution, followed by centrifugation. The supernatant was transferred to a clean 2-ml tube, 650 μL of isopropanol (Sigma-Aldrich®, St. Louis, MO, United States) was added, and the tube was centrifuged. The supernatant was poured off, and the tubes dried for 5 min, then the pellet was eluted with 95 μL of elution buffer (EB) (MinElute Gel extraction kit-Qiagen, Hilden, Germany). Approximately 30 μL of 8 M lithium chloride (Sigma, St. Louis, MO, USA) was added to the RNA, incubated at 4°C overnight for precipitation, and then centrifuged at 16000 *x g* at 4°C for 30 min. The mixture was purified using the MinElute gel extraction kit (Qiagen, Hilden, Germany) according to the manufacturer’s instructions. The purified product was verified by electrophoresis in a 1.0% agarose gel under ultraviolet light (Bio-Rad Laboratories, Hercules, CA, United States).

### 2.3. Complementary DNA synthesis and purification

Thirteen microliters of the purified dsRNA were used as a template for Complementary DNA (cDNA) synthesis using the Maxima H Minus Double-Stranded cDNA Synthesis Kit (Thermo Fischer Scientific, Waltham, MA) as described by the manufacturer protocol. Briefly, the dsRNA was denatured at 95°C for 5 min, then 1 μL of 100 μM of random hexamer primer was added to the denatured dsRNA. Afterward, there was a 5-min incubation in a thermocycler at 65°C. Following incubation, first-strand cDNA synthesis was performed by adding the First Strand Reaction Mix (5 μL) and the first-strand enzyme mix (1 μL). The tubes were incubated at 25°C for 10 min, followed by 2 h at 50°C. The reaction was terminated by heating at 85°C for 5 min. Following termination, 55 μL of nuclease-free water, 20 μL of 5X Second Strand Reaction Mix, and 5 μL of Second Strand Reaction Enzyme were added to the solution and incubated at 16°C for 60 min. The reaction was then stopped by adding 6 μL of 0.5 M EDTA and 10 μL of RNAse. The synthesized cDNA was incubated for 5 min at room temperature. Thereafter, the cDNA was purified using the MSB® Spin PCRapace Purification Kit (Stratec Molecular, Berlin, Germany).

### 2.4. DNA library preparations and whole genome sequencing

The DNA library for whole genome sequencing was prepared using the Nextera® XT DNA Library Preparation Kit (Illumina, Inc., San Diego, CA, United States), as described previously (Mwangi et al., 2020). The DNA library clean-up was conducted using AMPure XP magnetic beads (Beckman Coulter, Pasadena, CA, United States) and 80% ethanol. Quantitative and qualitative library validation was performed using the Qubit 3.0 fluorometer (Invitrogen, Carlsbad, CA, United States) and the Agilent 2,100 BioAnalyzer® (Agilent Technologies, Waldbronn, Germany), respectively. Whole genome sequencing was carried out using the Illumina MiSeq® sequencer (Illumina, Inc., San Diego, CA, USA), as previously described (Strydom et al., 2019; Mwangi et al., 2020; Rasebotsa et al., 2021).

### 2.5. Genome assembly

The raw Fastq sequence data were trimmed and then, *de novo*, assembled using CLC Bio Genomics Workbench (v.22.0) software^2^ to obtain the full-length nucleotide sequence of each genome segment. Reference mapping was employed to generate consensus sequences for all analyzed strains in Geneious Prime® (v.2022.0.1) software (Kearse et al., 2012) as a complementary tool.

### 2.6. Full-genome constellation analysis and genotype assignment

The resulting consensus sequences of all strains were used as query sequences to determine the genotypes of each of the 11 segments in the online database, Virus Pathogen Database and Analysis Resource (ViPR) (Pickett et al., 2012), and the full-length nucleotide sequence was determined using Nucleotide Basic Local Alignment Search Tool (BLASTn^3^) and Virus Variation Resource (Hatcher et al., 2017).

### 2.7. Phylogenetic analysis

The complete sequences for each gene segment of the study strains and reference sequences selected from GenBank were joined in a data set and aligned using Multiple Sequence Comparison by Log Expectation (MUSCLE) for each genome segment. Following sequence alignment, the evolutionary model that best fits each gene segment was determined. Based on the corrected Akaike information, the models identified to best fit the sequences were Tamura 3-parameter (T92) + gamma distributed (G) for VP7, VP4, VP6, NSP2, and NSP5, T92 + G+ invariable sites (I) (VP3), Tamura-Nei (TN93) + G + I (VP2), T92 + I (NSP1, NSP3-NSP4), and General Time Reversible (GTR) + G + I (VP1). The models were used to construct phylogenetic trees using Molecular Evolutionary Genetics Analysis (MEGA), version 11 (MEGA XI) (Tamura et al., 2021), applying the Maximum Likelihood method to infer for each segment. Tree topology robustness was evaluated through the bootstrapping method with 1,000 random samples. Bootstrap values equal to or greater than 70% of the groupings were considered consistent in the phylogenetic analysis. Pairwise distance matrixes for nucleotide and amino acid sequences were calculated using the p-distance algorithm in MEGA XI (Tamura et al., 2021).

## 3. Results

### 3.1. Genome assembly and constellations

A whole genome analysis was performed for the two G3P[8] strains. The strains were assigned as RVA/Human-wt/MOZ/MAN-1811463.8/2021/G3P[8] and RVA/Human-wt/MOZ/MAN-1811450.8/2021/G3P[8], with 722,344 and 937,370 reads after trimming, respectively. Their full- or near-full-length reads were assembled in all 11 segments. Complete open reading frames (ORFs) (100%) were obtained for 10 of the genome segments of each strain and partially (99%) for NSP1 in both strains (Table 1). The two strains revealed a Wa-like genotype constellation (G3P[8]-I1-R1-C1-M1-A1-N1-T1-E1-H1) (Table 1). All ORF sequences of the 11 genes of the two strains from Mozambique were deposited in GenBank under the accession numbers OQ398189–OQ398210.

**Table 1:** Whole genotype constellations and open reading frames (ORFs) of the 11 segments of G3P[8] strains from Manhiça District, Mozambique.

Strain nomenclature	Vaccine status													Genotype Constellation
		Gene segment	VP7	VP4	VP6	VP1	VP2	VP3	NSP1	NSP2	NSP3	NSP4	NSP5/NSP6	
RVA/Human-wt/MOZ/MAN-1811463.8/2021/G3P[8]	Vaccinated	Genome constellation	G3	P[8]	I1	R1	C1	M1	A1	N1	T1	E1	H1	Wa-like
		Contig length	1063.0	2350.0	1356.0	3302.0	2735.0	2620.0	1567.0	1059.0	1074.0	750.0	664.0	
		% Length	100.0	99.6	100.0	100.0	100.0	100.0	100.0	100.0	100.0	100.0	100.0	
		Average coverage	2143.0	8631.0	5754.0	24588.0	7197.0	7641.0	9460.0	3890.0	2312.0	1227.0	287.0	
		ORF length	981.0	2328.0	1217.0	3267.0	2707.0	2557.0	1492.0	1000.0	967.0	569.0	615	
		% ORF	100.0	100.0	100.0	100.0	100.0	100.0	99.0	100.0	100.0	100.0	100.0	
		% Identity	98.9	99.2	99.3	99.2	99.2	99.0	98.9	98.9	99.2	99.7	98.9	
		GenBank accession no. of the similar strain	MN836863	LC477393	MN414272	MZ096337	OL906392	OL906391	OL906389	MN304729	MN304730	KX6386 69	MN304732	
RVA/Human-wt/MOZ/MAN- 1811450.8/2021/G3P[8]	Vaccinated	Genome constellation	G3	P[8]	I1	R1	C1	M1	A1	N1	T1	E1	H1	Wa-like
		Contig length	1063.0	2359.0	1356.0	3302.0	2735.0	2591.0	1567.0	1059.0	1074.0	750.0	664.0	
		% Length	100.0	100.0	100.0	100.0	100.0	100.0	100.0	100.0	100.0	100.0	100.0	
		Average coverage	2643.0	1171.0	8188.0	30597.0	12311.0	10654.0	13234.0	4866.0	3515.0	2085.0	802.0	
		ORF length	1029.0	2328.0	1217.0	3267.0	2707.0	2557.0	1492.0	1000.0	967.0	569.0	615.0	
		% ORF	100.0	100.0	100.0	100.0	100.0	100.0	99.0	100.0	100.0	100.0	100.0	
		% Identity	98.7	99.2	98.7	99.2	99.2	99.1	99.8	99.1	99.3	99.7	99.2	
		GenBank accession no. of the similar strain	MN836863	LC477393	MN414272	MZ096337	OL906392	OL906391	OL906389	MN304729	MN304730	KX6386 69	LC600852	

The nomenclature of the strains indicates the rotavirus group, species where the strain was isolated, name of the country where the strain was originally isolated, common name, year of isolation, and genotypes for genome segments 4 and 9, as proposed by the Rotavirus Classification Working Group (RCWG) [6]. The length and percentage of ORF were determined by reference mapping using different strains with high identities obtained using the Basic Local Alignment Searching Tool (BLAST). % Identity-obtained from the BLAST.

### 3.2. Sequence and phylogenetic analysis

#### 3.2.1. Phylogenetic analysis of VP7 and VP4

**Figure 1:**
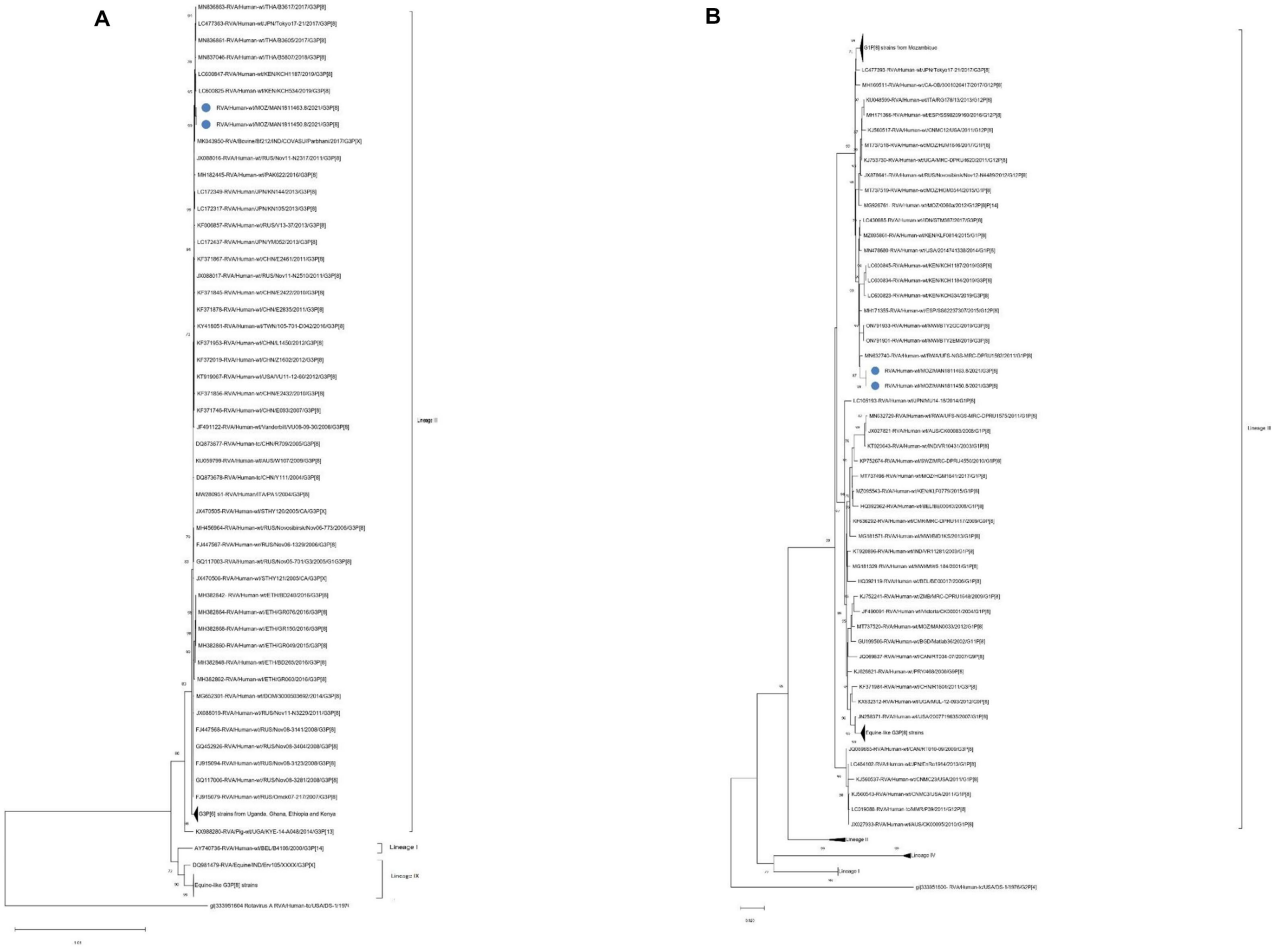
Phylogenetic trees of VP7 **(A)** and VP4 **(B)** based on the open reading frame (ORF) nucleotide sequences of RVA/Human-wt/MOZ/MAN-1811463.8/2021/G3P[8] and RVA/Human-wt/MOZ/MAN-1811450.8/2021/C3P[8] strains compared with global strains available from the Genebank. Manhiça strains are indicated by a filled blue circle symbol. Bootstrap values lower an 70% are not shown and the DS-1 like RVA strain from USA was serving as an out-group. Scale bars indicate the number of of substitutions per nucleotide position.

The VP7 genes of the Mozambican G3P[8] strains, RVA/Human-wt/MOZ/MAN-1811463.8/2021/G3P[8] and RVA/Human-wt/MOZ/MAN-1811450.8/2021/G3P[8] were compared with available sequences from GenBank representing previous established G3 lineages (I-III, IX) (Green Kim et al., 1988; Hatcher et al., 2017; Katz et al., 2019; Tamura et al., 2021), and they clustered into lineage III. Both Mozambican strains clustered closely together and shared 100% nucleotide (nt) and amino acid (aa) identities. Additionally, when compared to the closest strains in the cluster, they shared an average of 98.7% nt and 97.3% aa identities with Kenyan strains (RVA/Human-wt/KEN/KCH534/2019/G3P[8] and RVA/Human-wt/KEN/KCH1187/2019/G3P[8]) and 98.8% nt and 97.3% aa identities with a bovine strain from India (RVA/Bovine_Bf212/COVASU/Parbhani/2017_G3P[X]) (Figure 1A). Moreover, the G3 strains from the study clustered distinctly from the well described and emerging equine-like G3 strains from the Dominican Republic, Australia, Thailand, Spain, and Japan with an overage of 78.0% nt and 68.8% aa identities (Figure 1A and Supplementary Table S1).

Meanwhile, the VP4 genes of the two strains were compared with human rotavirus sequences previously known from four lineages (I-IV) (Dormitzer, 2002; Dormitzer et al., 2004; Monnier et al., 2006), and they clustered within lineage III and were closely related to a strain from Rwanda (RVA/Human-wt/RWA/UFS-NGS-MRC-DPRU1582/2011/G1P[8]), Kenyan strains (RVA/Human-wt/KEN/KCH1184/2019/G3P[8] and RVA/Human-wt/KEN/KCH1187/2019/G3P[8]) with an average of 98.9% nt and 99.1% aa identities. Conversely, both strains formed distant clusters from Mozambican strains circulating from 2012 to 2017, and specifically from local strains from Manhiça, before vaccine introduction, RVA/Human-wt/MOZ/0060a/2012/G12P[8]P[14] and RVA/Human-wt/MOZ/MAN0033/2012/G1P[8]. Additionally, the study strains clustered distantly from P[8] of the emerging G3 DS-1-like strains (Figure 1B and Supplementary Table S1).

#### 3.2.2. Comparative analysis of the deduced amino acids of the variable regions of Mozambican G3 (VP7) strains and G3 bovine, equine, and other wild-type strains

**Figure 2:**
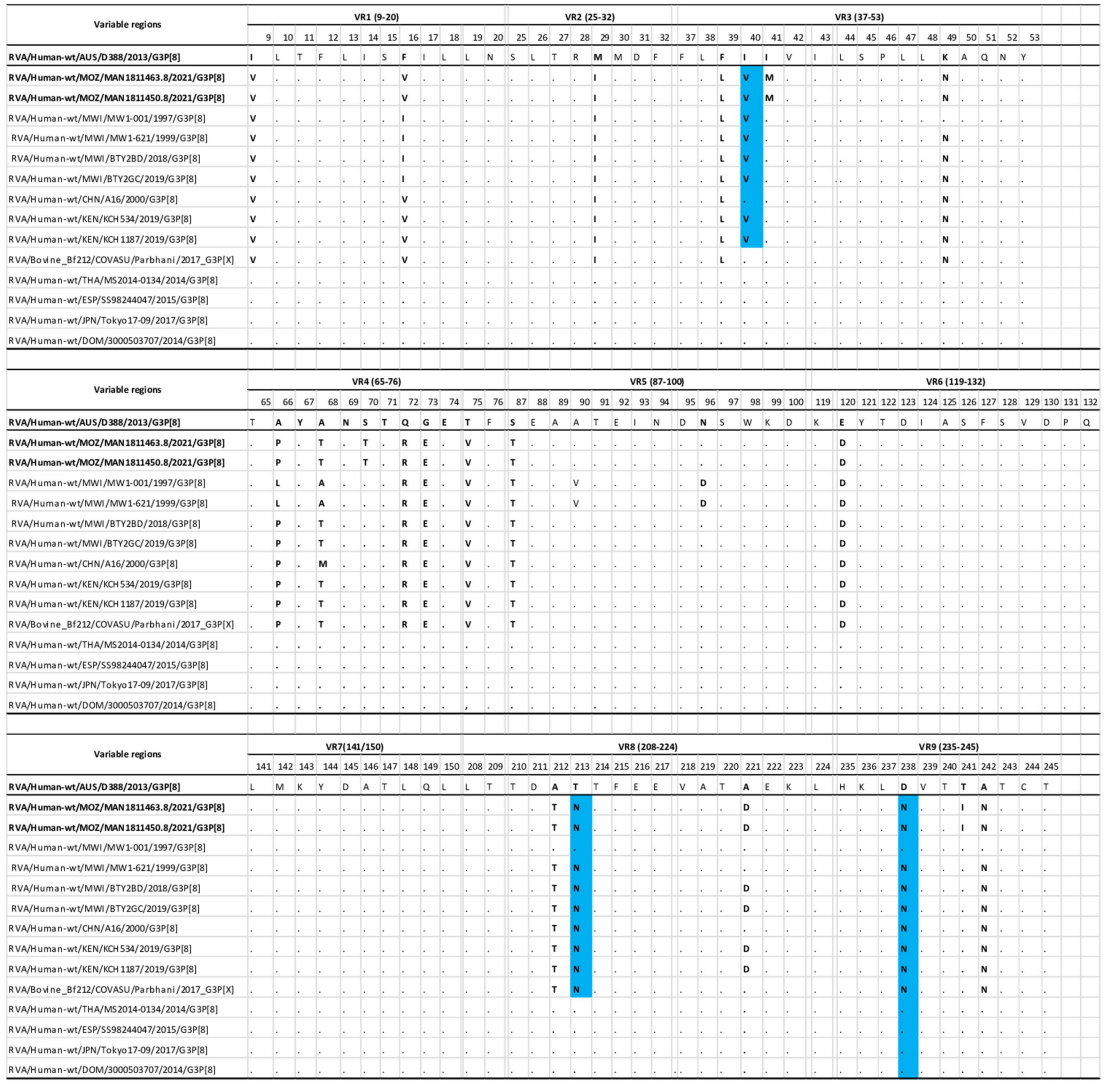
Comparison of the deduced amino acid of VP7 gene of the G3 genotype of the study strains, with wild type strains (MWI-001, MW 1–621, BTY2BD. BTY2GC, CHN/A16, KCH534, KCH1187), G3 bovine (Bf212/COVASU/Parbhani) and selected emerging G3P[8] equine-like strains (SS98244047, MS2014-0134, Tokyo17-09, 3000503707). The D388 equine strain from Australia is the reference strain. Conserved residues between the reference and the comparative strains are indicated in black dots (.) and residues differing between them, are indicated with the bolded letter. Blue colored residues represents amino acids substitutions, which may have effects on the immunogenicity.

The deduced amino acid sequences of the VP7 of the study strains were compared with equine-like and other G3P[8] strains from pre- and post-vaccine introduction periods, including the bovine strain, using previously described variable regions (VR) 1–9 (Green Kim et al., 1988). The prototype G3 equine strain from Australia was used as consensus because of the recent emergence of G3 equine strains worldwide. Overall, multiple amino acid differences were observed between the variable regions in the study strains and the equine-like strains in 21 of a total of 115 positions. These variable regions were concentrated in regions 3, 4, and 9 (Figure 2). Three important amino acid changes were I40V, T213N, and D238N (Figure 2). There were slight differences observed between the study strains and the other wild-type G3P[8], bovine, and G3-equine-like strains. These differences were observed in regions I41-M, S70-T, and T241I (Figure 2).

#### 3.2.3. Comparative analysis of neutralizing antigenic epitopes of VP4 genes of the study strains with Rotarix® vaccine strains, equine strains, and other wild-type strains

**Figure 3:**
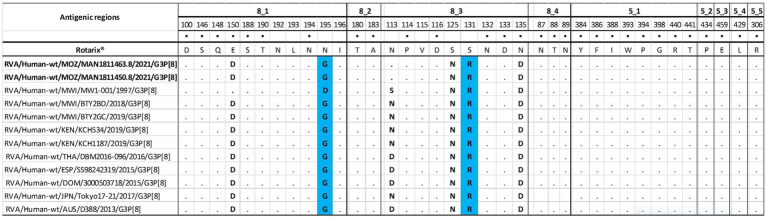
Alignment of P[8] amino acids corresponding to antigenic epitopes (8-1, 8-2, 8-3, 8-4-VP8*subunit and 5-1, 5-2, 5-3, 5-4, 5-5- VP5* subunit) of VP4 of the study strains, with wild type P[8] from Malawi (MW1-001, BTY2BD, BTY2GC), Kenyan (KCH534 and KCH1187) and selected P[8] from the emerging G3P[8] equine-like strains (DBM2016-096, SS98242319, 3000503718, Tokyo17-21 and D388). Rotarix® is the reference strain. The black dot indicates changes in the amino acids that have been demonstrated to escape neutralization with monoclonal antibodies. The conserved residues between the reference and the comparative strains are indicated in black dots (.) and residues differing between them are indicated with the bolded letter. Blue colored residues represents amino acids substitutions that may escape host-immunity.

The VP4 protein activation is mediated by cleavage of its two subunits, the VP8* region, which contains four (8–1 to 8–4) neutralizing antigenic epitopes, and the VP5* region, which has five (5–1 to 5–5) (Dormitzer, 2002; Dormitzer et al., 2004). The two epitopes have 37 amino acid residues, of which 25 are in the VP8* and 12 in the VP5* (Monnier et al., 2006). The study strains, wild type, and equine strains were identical to Rotarix® strains in 32 of 37 amino acid residues and different in positions (E150D, N195G, S125N, S131R, and N135D). The differences between the study strains and the emerging G3P[8] equine-like strains were in position 113 in the 8–3 epitope, a switch from an asparagine (N) to an aspartic acid (D) (Figure 3).

#### 3.2.4. Phylogenetic analyses of VP1–VP3 and VP6

The two Mozambican strains clustered together in a monophyletic cluster in all the trees (VP1–VP3), sharing 100% nt and aa identities among them, except in the VP6 gene segment, in which they were similar in 99.2% nt and 97.2% aa composition. Singularly, analysis of the VP1 encoding segment revealed that the study strains grouped in a major cluster containing human and animal strains, with an average of 91.3% nt and 97.2% aa identities with porcine and 86.9% nt and 97.6% aa with equine strains; however, they were closely related to G3P[8] Kenya strains with an average of 99.2% nt and 99.7% aa identities (Supplementary Table S1 and Supplementary Figure S2A). The VP2 clustered near human strains from Mozambique, which circulated from 2014 to 2017, and strains from Thailand, India, Malawi, the Dominican Republic, Rwanda, and Kenya, while the VP3 was closely related to locally circulating strains from Manhiça and several strains from Kenya, the United States, and Japan. In all the trees of the encoding gene segments VP1–VP3, the study strains were distant from strains locally circulating in Manhiça in 2012, RVA/Human-wt/MOZ/0042/2012/G12P[6], RVA/Human-wt/MOZ/0060a/2012/G12P[8]P[14] and RVA/Human-wt/MOZ/MAN0033/2012/G1P[8] (Supplementary Figures S2A–C). Conversely, VP6 presented a different pattern, showing a more close relationship to some groups of strains from Japan, India, Kenya, Malawi, and a porcine strain from Mozambique, RVA/Pig-wt/MOZ/MZ-MPT-200/2016/G9P[13], with 98.3%nt and 97.5% aa identities (Supplementyary Table S1 and Supplementary Figure S2D).

#### 3.2.5. Phylogenetic analyses of the NSP1-NSP5

The NSP1–NSP5 genes of the study strains formed a cluster that was closely related to each other, sharing absolute (100%) nt and aa identities. The NSP1 clustered with some Mozambican strains that had been reported from 2012 to 2015, two of them locally circulating in Manhiça (RVA/Human-wt/MOZ/0289/2012/G12P[6] and RVA/Human-wt/MOZ/0042/2012/G12P[6]) and strains from Spain and Kenya. While NSP2 clustered with strains from Rwanda, the Dominican Republic, and Benin, and a strain from Mozambique (RVA/Human wt/MOZ/HJM1646/2017/G1P[8]). Both NSP1 and NSP2 encoding segments were revealed to be distant from some strains circulating locally in Manhiça (RVA/Human-wt/MOZ/0042/2012/G12P[6] and RVA/Human-wt/MOZ/0060a/2012/G12P[8]P[14]; Supplementary Figures S2E,F). On the other hand, NSP3 clustered near human Mozambican and Rwandan strains, porcine strains from China with an average of 99.0% nt and 97.6% aa identities, and a bovine strain from Uganda with 99.4% nt and 98.6% aa identities (Supplementary Table S1 and Supplementary Figure S2G). Moreover, NSP4 clustered with human rotavirus strains from China and Japan and with porcine strains from Mozambique (RVA/Pig-wt/MOZ/MZ-MPT-195/2016/G9P[13] and RVA/Pig-wt/MOZ/MZ-MPT-200/2016/G9P[13]), with an average of 99.9% nt and 100.0% aa identities, and a bovine strain from the United Kingdom with 97.4% nt and 98.9% aa identities (Supplementary Table S1 and Supplementary Figure S2H). NSP5/6 clustered with Kenyan, Malawian, and Indian strains and a strain locally circulating in Manhiça (RVA/Human-wt/MOZ/MAN0033/2012/G1P[8]) but was distant from a strain from Manhiça (RVA/Human-wt/MOZ/0060a/2012/G12P[8]P[14]) and other Mozambican strains circulating from 2014 to 2017 (Supplementary Table S1 and Supplementary Figure S2I).

## 4. Discussion

This is one of the few studies that sequenced rotavirus G3P[8] strains by WGS in Mozambique. The results revealed that the G3P[8] study strains were of genotype one constellation (Wa-like), and phylogenetic analysis of all 11 genes demonstrated that they were closely related to each other. The VP7 (G3) and VP4 (P[8]) clustered in lineage III. The two study strains consistently formed distinct clusters with some human strains, circulating from 2012 to 2019 in Africa (Mozambique, Kenya, Rwanda, Malawi, Congo, Togo, Uganda), Asia (Japan, China, and India), North and South America (United States and Dominican Republic), and Europe (Italy and Russia) in six genome segments (VP2, VP3, NSP1-NSP2, NSP5/6). G3P[8] strains have been spreading after introducing the rotavirus vaccine in many countries (Roczo-Farkas et al., 2018; João et al., 2020; Mwanga et al., 2020; Bonura et al., 2022; Manjate et al., 2022), so studying its worldwide evolution is still essential. Previous studies reported the full genome sequence of G3 strains from different regions of the world and demonstrated that it was a new equine-like strain, frequently associated with the genotype two constellations (DS-1-like) (Cowley et al., 2016; Dóró et al., 2016; Cowley et al., 2018; Pietsch and Liebert, 2018; Esposito et al., 2019; Katz et al., 2019; Tacharoenmuang et al., 2019; Amit et al., 2021; Gutierrez et al., 2021; Martinez-Gutierrez et al., 2021), contrary to the strains in this study, which are of genotype 1, and clustered distantly from the equine-like strains. This fact may, in part, suggest that the two G3P[8] study strains may have arisen by importing strains from other countries. Additionally, unlike our study, previous sequence analysis of G3 strains from Kenya after the rotavirus vaccine introduction showed that they fit within lineages I and IX (the latter, also known as the equine-like G3 lineage) (Mwanga et al., 2020). Wa-like G3 strains from Italy fit into lineage I, and their P[8] were from lineage III (Medici et al., 2016).

Considering the antigenic regions of VP7, which are important for serotype antigenic variation (Green Kim et al., 1988), when comparing the equine G3 from Australia with the studied strains, it was observed that they exhibited an amino acid change of D238N. In equine-like G3 strains, this position is described as being associated with the loss of a potential glycosylation site (Kirkwood et al., 1993), which determines viral immunogenicity by modulating virus receptor binding or masking antigenic sites (Caust et al., 1987; Mao et al., 2022). A study with monoclonal antibodies (mAB) has shown that mAb F45:2 of the equine-like G3P[8] strains bind to G3 and G9 strains that lack this suspected glycosylation site (Kirkwood et al., 1993). Amino acid changes seen at positions A212T, T213N, A221D, and D242N of the study strains have been previously described in Belgium (Zeller et al., 2012). The substitution at position 213, with or without glycosylation changes, is known to alter the antigenicity of viruses (Ahmed et al., 2007) and may be important in antigenic drift, escaping host immunity (Maranhão et al., 2012). An important change was observed at position 40, from isoleucine to valine. The isoleucine substitution with valine is described to increase electrophoretic mobility and to be associated with the shift of the protein equilibrium to a more compact conformation (Keating and Cronan, 1996).

Additionally, some amino acid changes occurred only in the study strains, such as the I41M substitution and are hydrophobic amino acids, which are not implied in the protein functionality but are associated with the binding/recognition of hydrophobic ligands such as lipids (Betts and Russell, 2003). The S70T substitution involves both polar amino acids, which can be fairly reactive (Betts and Russell, 2003). And T241I, a polar amino acid that is common in functional protein centers, to a non-polar amino acid without functional activities (Betts and Russell, 2003). Several other amino acid differences were observed between the study strains and the equine-like G3 strains. This has also been reported in previous analyses of wild-type G3P[8] strains from Australia, suggesting that the equine-like strains may be antigenically distinct from human strains (Cowley et al., 2016). Different patterns of reactivity to VP7-specific monoclonal antibodies between equine-like and wild-type G3 strains were previously observed (Cowley et al., 2016). However, it is not clear how these differences have contributed to the emergence of these new equine-like G3 strains (Bonura et al., 2021).

However, the VP4 antigenic epitopes exhibited conserved substitutions in the positions E150D, S125N, and N135D among the study strains when compared to the Rotarix® strain and a pre-vaccine strain from Malawi included in the analysis as a regional reference strain. However, VP4 presented changes at the positions N195G and S131R, which were described in previous studies to be associated with amino acid changes that may have resulted in polarity changes, which are important for the virus to escape host immunity (Rasebotsa et al., 2020; Maringa et al., 2021). These results may not be associated with the vaccine introduction, as the Malawian strain presented changes in the same positions. In addition, it was observed that the strains characterized formed a distant cluster from some locally circulating strains from Manhiça in the gene segments VP1-VP3, NSP1, and NSP2. One of the distant strains was isolated in 2012 (MAN0033/2012/G1P[8]) and was previously reported to be distant from other Mozambican G1P[8] strains from 2012 to 2017. However, it was shown to be closely related to a conserved group of Malawian strains, allowing us to speculate about a possible recent introduction in Mozambique from a neighboring country (Munlela et al., 2020). Moreover, the phylogenetic analysis demonstrated that VP7 clustered most closely with a bovine strain from India, VP6 with a Mozambican porcine strain, VP1 with porcine strains from Belgium, South Korea, Japan, and an equine strain from the United Kingdom, NSP3 with a porcine strain from China and a bovine strain from Ugandan and NSP4 with Mozambican porcine strains. Overall, the identities ranged from 86.9–99.9% nt and 97.2–100% aa, and the porcine strains were mainly G9 strains combined with P[7], P[13], and P[23].

A close relationship between rotavirus G9 porcine strains was previously reported in a large-scale whole-genome-based study of G3P[8] in China, in which NSP3 genes clustered with porcine-like human G9 strains. It was suggested that the G3 strains in this study may have acquired their NSP3 gene via intra-genotype reassortment and that this may have occurred from porcine or porcine-like human rotaviruses. If confirmed, it may be indicative of the possible persistence and potential spread of the gene segment of zoonotic origin among common genotypes of human rotavirus (Wang et al., 2014). This also stresses the need to study NSP3 genes from porcine rotaviruses (Wang et al., 2014). Furthermore, another study showed that the VP6 and NSP4 genes of a reassortant porcine-like G5P[6] human Wa-like strain had close similarity to porcine strains (Maringa et al., 2020), supporting the hypothesis that human Wa-like and porcine strains have a common ancestor (Matthijnssens et al., 2008).

Additionally, compared to other gene segments, NSP4 seems to display a higher evolutionary rate, and reassortment events were demonstrated to occur more frequently in this gene segment (Zeller et al., 2012; Tatsumi et al., 2014). A study analyzing a rare human G9P[3] AU-like strain found that the NSP4 gene segment resembled human G2P[4] (DS-1-like constellation) (Theamboonlers et al., 2013), suggesting a reassortment between AU-like and Kun-like strains (De Grazia et al., 2008). Rotavirus strains bearing unusual combinations of cross-species phenotype in humans and animals are increasingly detected and documented (Maneekarn et al., 2006; Dóró et al., 2014). These observations support the hypothesis of natural interspecies transmission of rotavirus strains, including to and from humans (Gouvea et al., 1990; Cook, 2004), resulting from an infection with animal strains or reassortment between human and animal strains (Martella et al., 2006).

However, it has been reported that whole rotavirus genotype segments of animal origin are not transmitted to humans. Transference of individual gene segments from other species to humans is the most likely and frequent event (Matthijnssens et al., 2010). During co-infection events, the gene segments of animal rotaviruses may reassort with human rotavirus segments, and this may result in human infection by reassortants whose part of the genome is of animal origin (Maestri et al., 2012). Events of infection by bovine and porcine strains in humans were reported to occur more frequently in rural areas, where humans are involved in rearing cattle and are in close proximity to animals (Midgley et al., 2012). The same condition was observed in the rural area of Manhiça (Manjate et al., 2022). One of the main limitations of this study was that it was only based on the analysis of two G3P[8] strains. It was not possible to perform reassortment analysis as the study strains, which are closely related to animal strains, have different combinations of VP7 and VP4 gene segments. The close relationship we demonstrated between some human rotavirus gene segments and those of various animal strains may suggest that reassortment events may have occurred between human and animal strains.

## 5. Conclusion

The similarities observed between the two strains characterized may indicate that they are clonally related. Additionally, the identification of segments exhibiting the closest relationships with animal strains shows significant diversity of rotavirus and may indicate that even with the high efficacy of rotavirus vaccines in reducing severe cases, rotavirus strains continue to evolve, and novel strains still emerge. Consequently, this highlights the importance of implementing molecular methods such as whole genome sequencing to monitor and understand the evolutionary changes of rotaviruses and evaluate the impact of vaccines on strain diversity. Additionally, our findings show the role of genome reassortment in driving rotavirus diversity and human mobility in disseminating rotavirus strains regionally.

## Data availability statement

Sequences obtained in the present study were deposited in GenBank (https://www.ncbi.nlm.nih.gov/genbank/) under the accession numbers OQ398189-OQ398210.

## Ethics statement

This study was reviewed and approved by Mozambican National Bioethics Committee, Ministry of Health, Mozambique. Written informed consent to participate in this study was provided by the participants’ legal guardian/next of kin.

## Author contributions

FM, MN, EJ, and IM: conceptualization. FM, EJ, MN, PM, and IM: methodology. FM, EJ, and PM: software. EJ and IM: validation. FM, PM, and EJ: formal analysis. FM: investigation. FM and EJ: resources, visualization, and project administration. FM, EJ, and IM: data curation and writing – original draft preparation. FM, PC, MG, DV, AM, SA, TN, NN, CC, MN, JT, UP, GW, PA, EJ, and IM: writing – review and editing. EJ, MN, and IM: supervision. IM, TN, PA, and JM: funding acquisition. All authors read and agreed to the published version of the manuscript.

## Funding

The GAVI, the vaccine alliance via the Centers for Disease Control and Prevention (CDC), Atlanta, and the World Health Organization, Regional Office for Africa (WHO/AFRO), grant number MOA #870–15 SC; the United States Agency for International Development (USAID), grant number AID-656-F-16-00002 and Fundo Nacional de Investigação (FNI), Moçambique, grant number 245-INV, funded the surveillance of rotavirus and other enteropathogens in children less than 5 years of age in Manhiça, in the context of the implementation of the diarrhoeal disease surveillance platform. The Child Health and Mortality Prevention program (Surveillance), CHAMPS funded by the Bill & Melinda Gates Foundation under Grant OPP1126780, the Instituto de Higiene e Medicina Tropical (IHMT), Universidade Nova de Lisboa, Portugal, the Next Generation Sequencing Unit, and the Division of Virology, Faculty of Health Sciences, University of the Free State, South Africa, supported the whole genome analysis costs. The Calouste Gulbenkian Foundation finances Filomena Manjate’s Ph.D. studies under grant number 234066. CISM receives core funding from the Mozambican government and the “Agencia Española de Cooperacion Internacional para el Desarollo (AECID).”

## Conflict of interest

The authors declare that the research was conducted in the absence of any commercial or financial relationships that could be construed as a potential conflict of interest.

## Publisher’s note

All claims expressed in this article are solely those of the authors and do not necessarily represent those of their affiliated organizations, or those of the publisher, the editors and the reviewers. Any product that may be evaluated in this article, or claim that may be made by its manufacturer, is not guaranteed or endorsed by the publisher.

## Author disclaimer

The findings and conclusions in this report are those of the authors and do not necessarily represent the official position of the Centers for Disease Control and Prevention.

## Footnotes

^1^ https://rega.kuleuven.be/cev/viralmetagenomics/virus-classification/rcwg

^2^ https://www.qiagenbioinformatics.com

^3^ https://blast.ncbi.nlm.nih.gov/
