# Supplementary material for: Genomic characterization of the rotavirus G3P[8] strain in vaccinated children, reveals possible reassortment events between human and animal strains in Manhiça District, Mozambique
Source: Front Microbiol. 2023 Jun 5;14:1193094. doi: 10.3389/fmicb.2023.1193094 (PMC10277737; doi:10.3389/fmicb.2023.1193094)
Supplement: Supplementary file 2 [file Data_Sheet_1.pdf]

Supplementary Material 2

Genomic characterization of rotavirus G3P[8] strain in vaccinated children, reveals possible reassortment events between human and animal strains in Manhiça District, Mozambique

Filomena Manjate[1,2], Eva D. João[1], Peter Mwangi[3], Percina Chirinda[1], Milton Mogotsi[3] Augusto Messa Jr.[1], Marcelino Garrine[1,2], Delfino Vubil[1], Nélio Nobela[1], Tacilta Nhampossa[1,4], Sozinho Acácio[1,4], Jacqueline E. Tate[5], Umesh Parashar[5], Goitom Weldegebriel[6], Jason M. Mwenda[6], Pedro Alonso[1,7], Celso Cunha[2] , Martin Nyaga[3] and Inácio Mandomando[1,4,7]*

[1] Centro de Investigação em Saúde de Manhiça (CISM), Maputo 1929, Mozambique; [2] Global Health and Tropical Medicine (GHTM), Instituto de Higiene e Medicina Tropical (IHMT), Universidade Nova de Lisboa (UNL), 1349-008 Lisbon, Portugal; [3] Next Generation Sequencing Unit and Division of Virology, Faculty of Health Sciences, University of the Free State, Bloemfontein 9300, South Africa; [4] Instituto Nacional de Saúde, Ministério da Saúde, Marracuene 1120, Mozambique; [5]Centers for Disease Control and Prevention (CDC), Atlanta, GA 30333, USA; [6]African Rotavirus Surveillance Network, Immunization, Vaccines and Development Program, World Health Organization, Regional Office for Africa, Brazzaville P.O. Box 2465, Congo; [7] ISGlobal, Hospital Clínic, Universitat de Barcelona, 08036 Barcelona, Spain

*** Correspondence:** inacio.mandomando@manhica.net

1 Supplementary figures

A. VP1

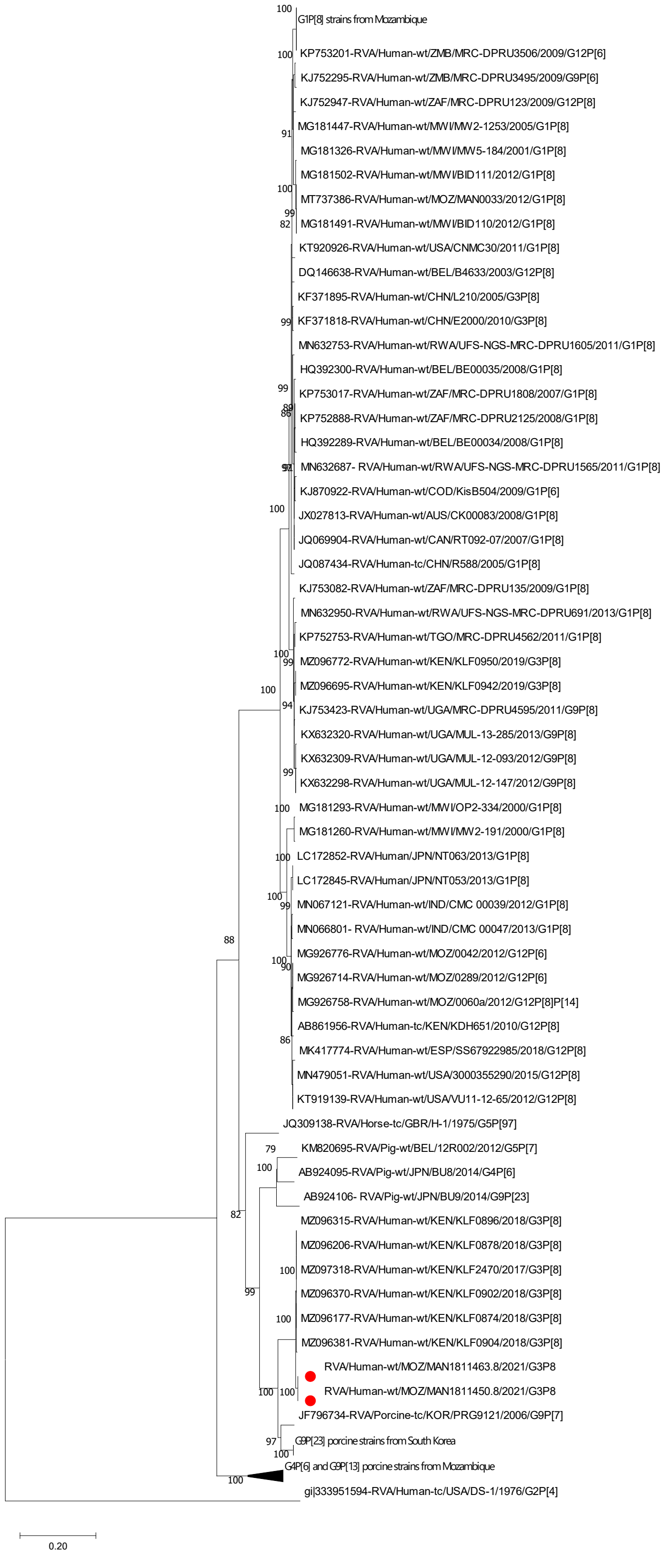

Supplementary Figure A: Phylogenetic tree of VP1 based on the open reading frame (ORF) nucleotide sequences of RVA/Human-wt/MOZ/MAN-1811463.8/2021/G3P[8] and RVA/Human-wt/MOZ/MAN-1811450.8/2021/G3P[8] strains compared with global strains available from the Genbank. Manhiça strains are indicated by a filled red circle symbol.

B. VP2

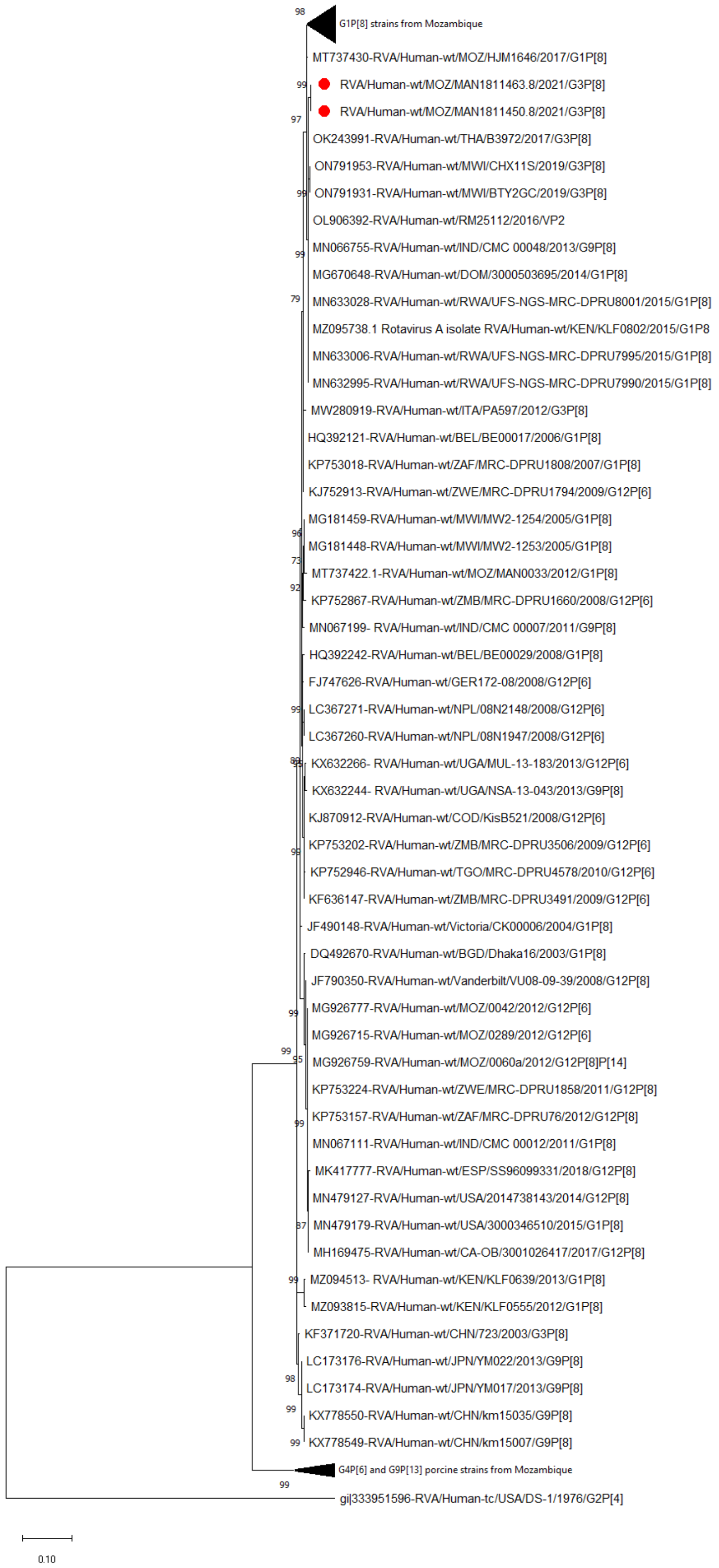

Supplementary Figure B: Phylogenetic tree of VP2 based on the open reading frame (ORF) nucleotide sequences of RVA/Human-wt/MOZ/MAN-1811463.8/2021/G3P[8] and RVA/Human-wt/MOZ/MAN-1811450.8/2021/G3P[8] strains compared with global strains available from the Genbank. Manhiça strains are indicated by a filled red circle symbol.

C. VP3

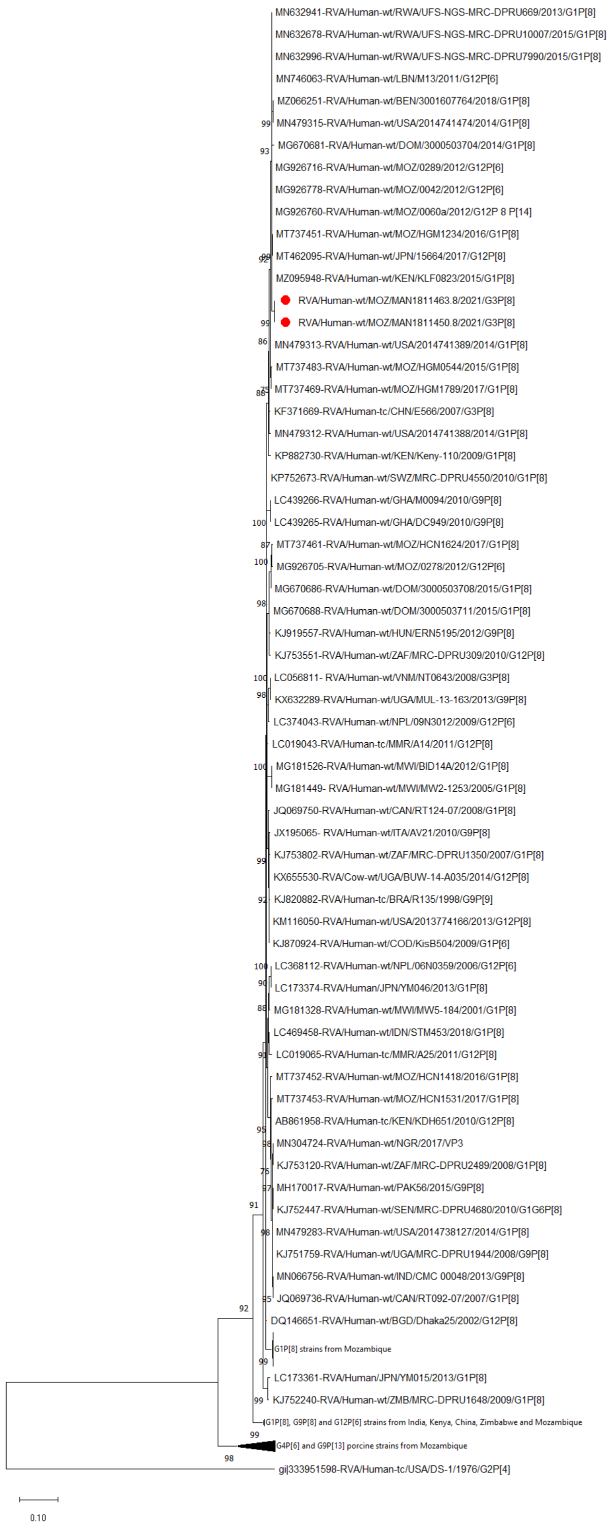

Supplementary Figure C: Phylogenetic tree of VP3 based on the open reading frame (ORF) nucleotide sequences of RVA/Human-wt/MOZ/MAN-1811463.8/2021/G3P[8] and RVA/Human-wt/MOZ/MAN-1811450.8/2021/G3P[8] strains compared with global strains available from the Genbank. Manhiça strains are indicated by a filled red circle symbol.

D. VP6

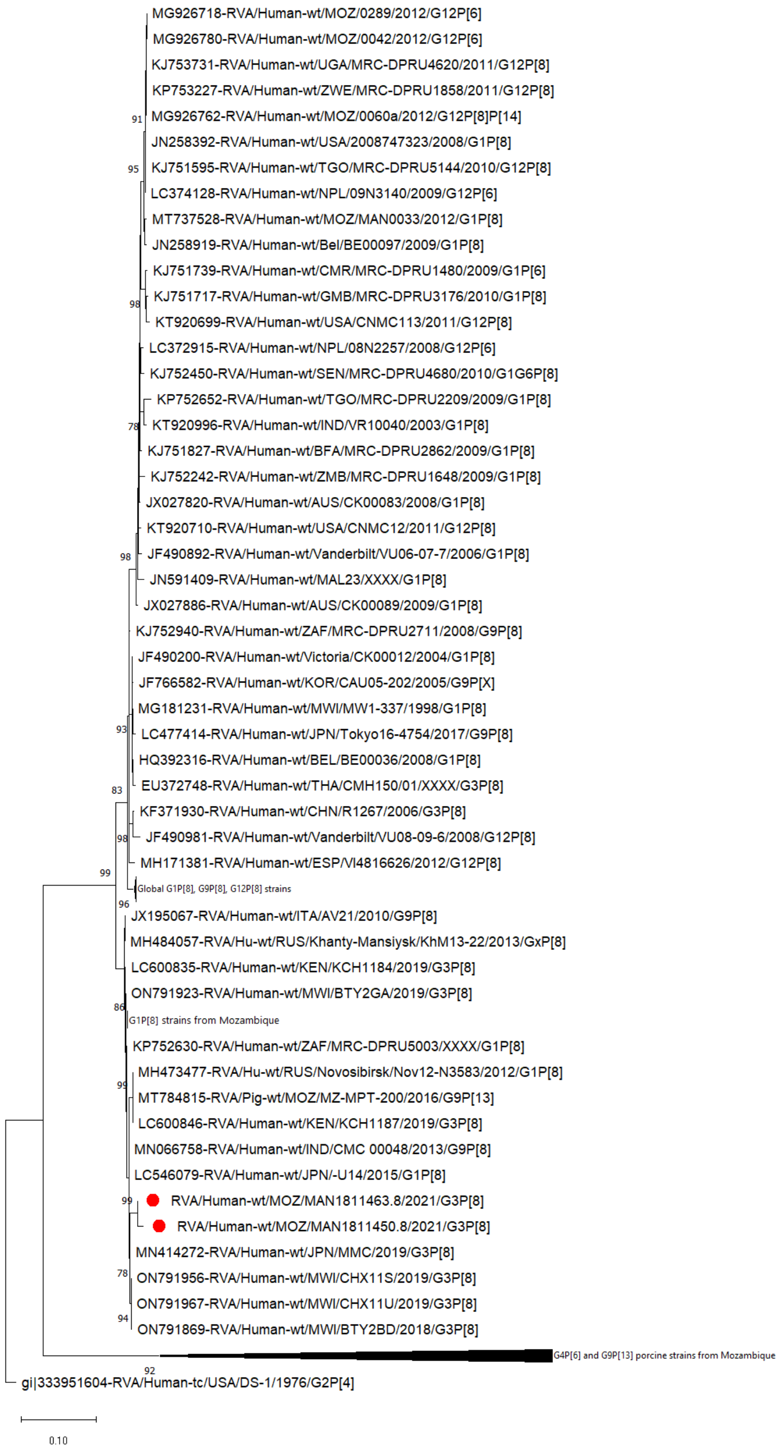

Supplementary Figure D: Phylogenetic tree of VP6 based on the open reading frame (ORF) nucleotide sequences of RVA/Human-wt/MOZ/MAN-1811463.8/2021/G3P[8] and RVA/Human-wt/MOZ/MAN-1811450.8/2021/G3P[8] strains compared with global strains available from the Genbank. Manhiça strains are indicated by a filled red circle symbol.

E. NSP1

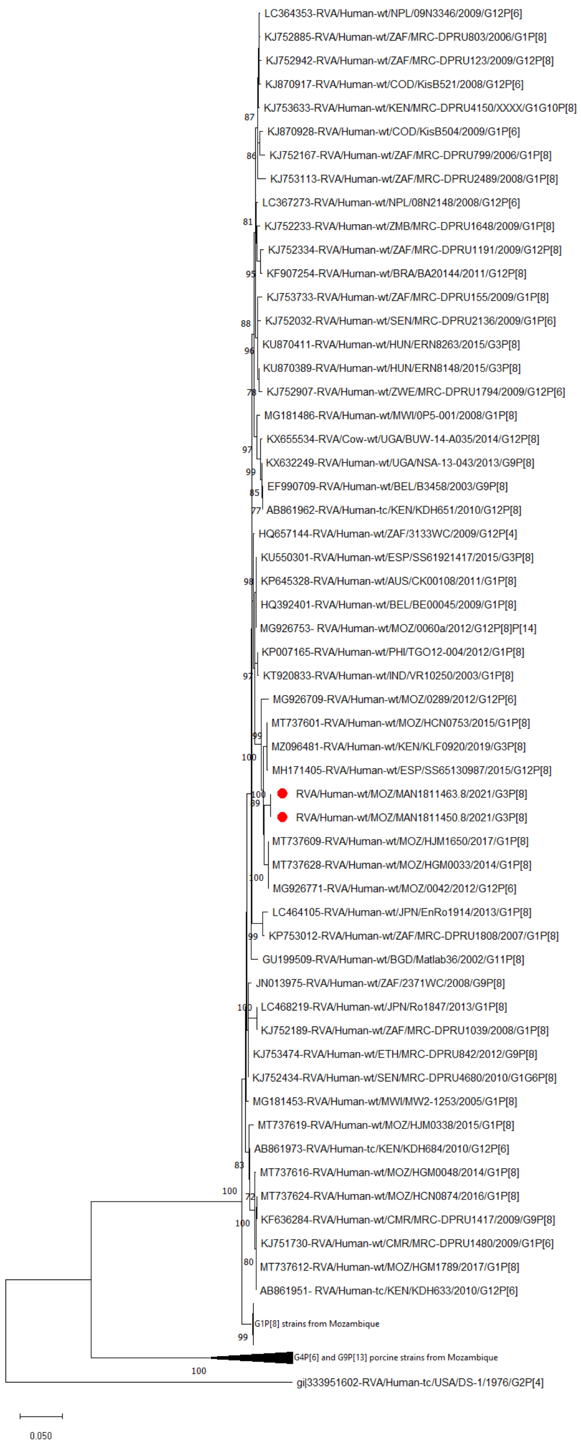

Supplementary Figure E: Phylogenetic tree of NSP1 based on the open reading frame (ORF) nucleotide sequences of RVA/Human-wt/MOZ/MAN-1811463.8/2021/G3P[8] and RVA/Human-wt/MOZ/MAN-1811450.8/2021/G3P[8] strains compared with global strains available from the Genbank. Manhiça strains are indicated by a filled red circle symbol.

F. NSP2

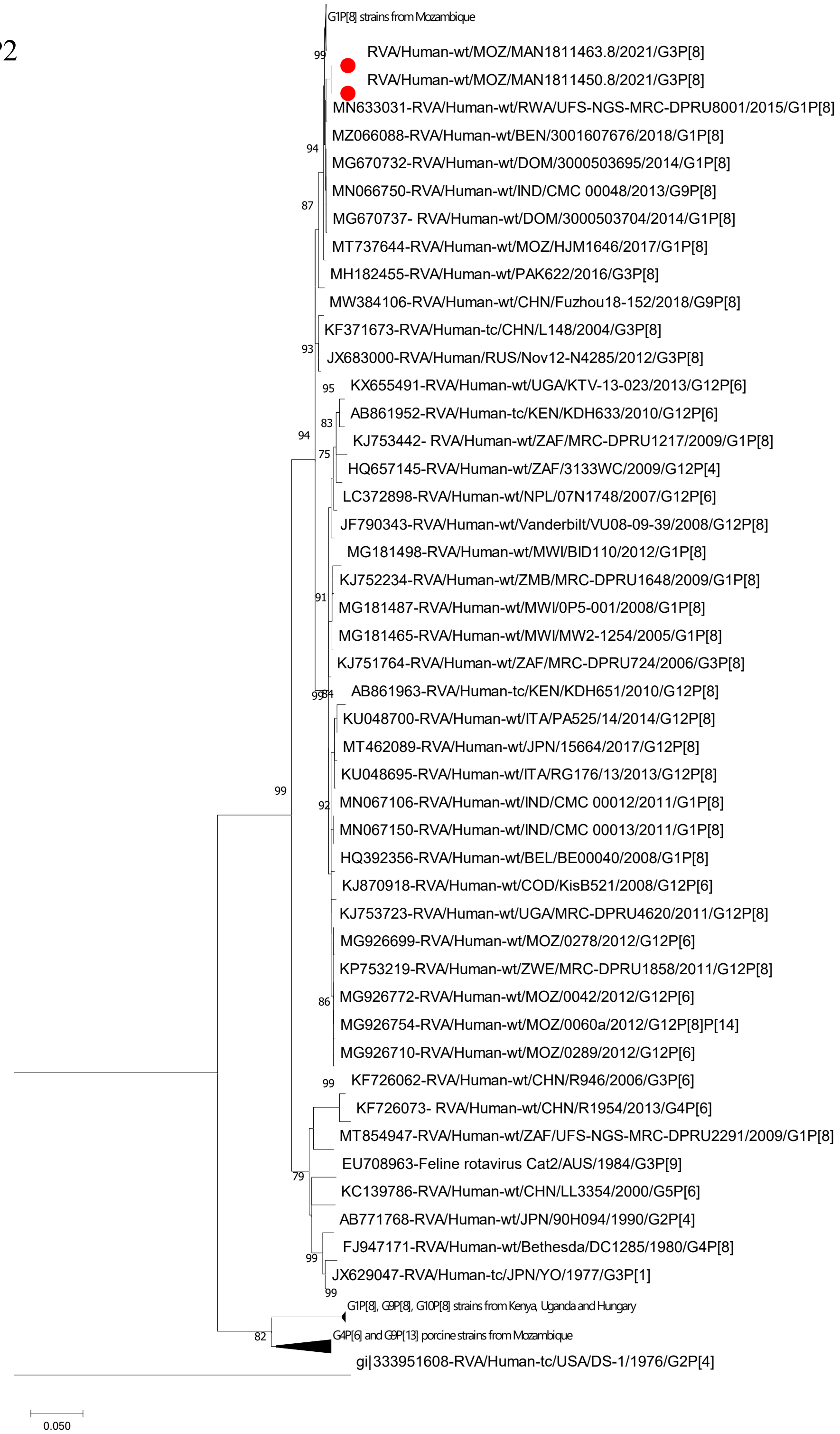

Supplementary Figure F: Phylogenetic tree of NSP2 based on the open reading frame (ORF) nucleotide sequences of RVA/Human-wt/MOZ/MAN-1811463.8/2021/G3P[8] and RVA/Human-wt/MOZ/MAN-1811450.8/2021/G3P[8] strains compared with global strains available from the Genbank. Manhiça strains are indicated by a filled red circle symbol.

G. NSP3

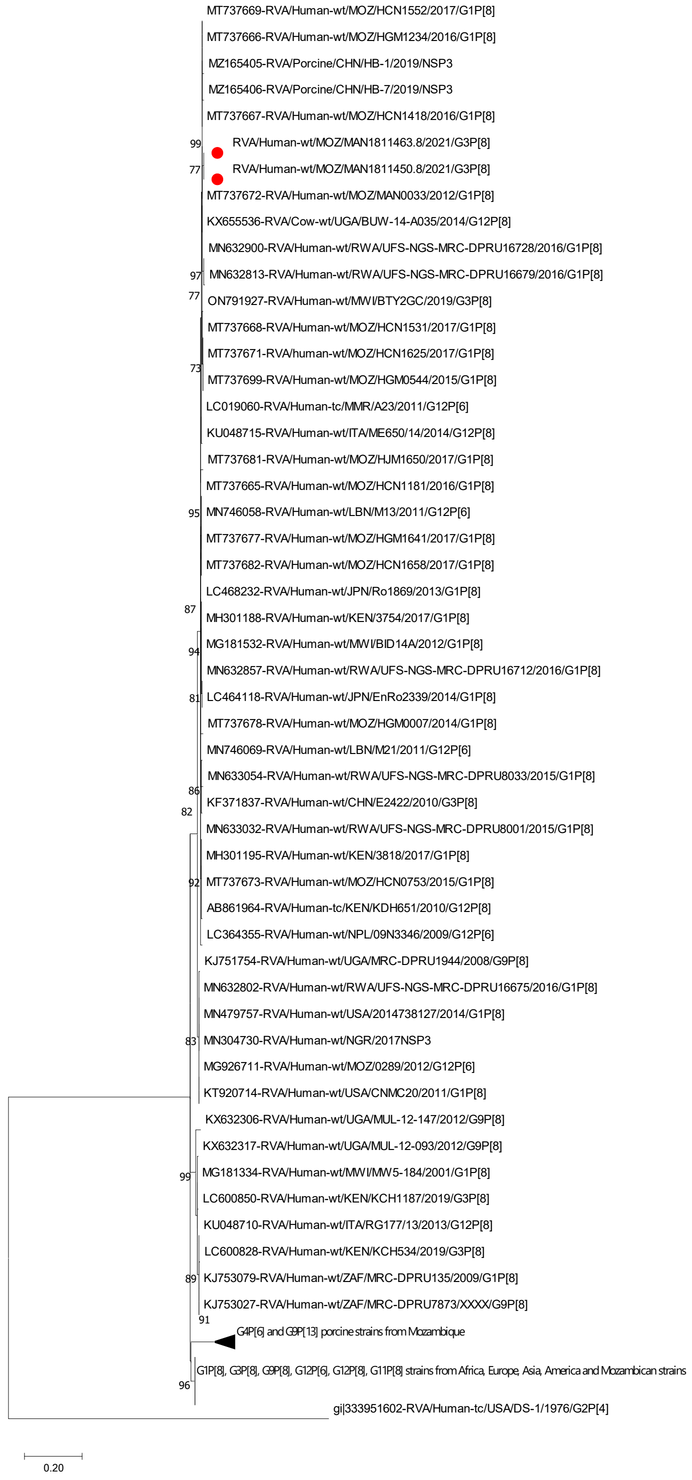

Supplementary Figure G: Phylogenetic tree of NSP3 based on the open reading frame (ORF) nucleotide sequences of RVA/Human-wt/MOZ/MAN-1811463.8/2021/G3P[8] and RVA/Human-wt/MOZ/MAN-1811450.8/2021/G3P[8] strains compared with global strains available from the Genbank. Manhiça strains are indicated by a filled red circle symbol.

H. NSP4

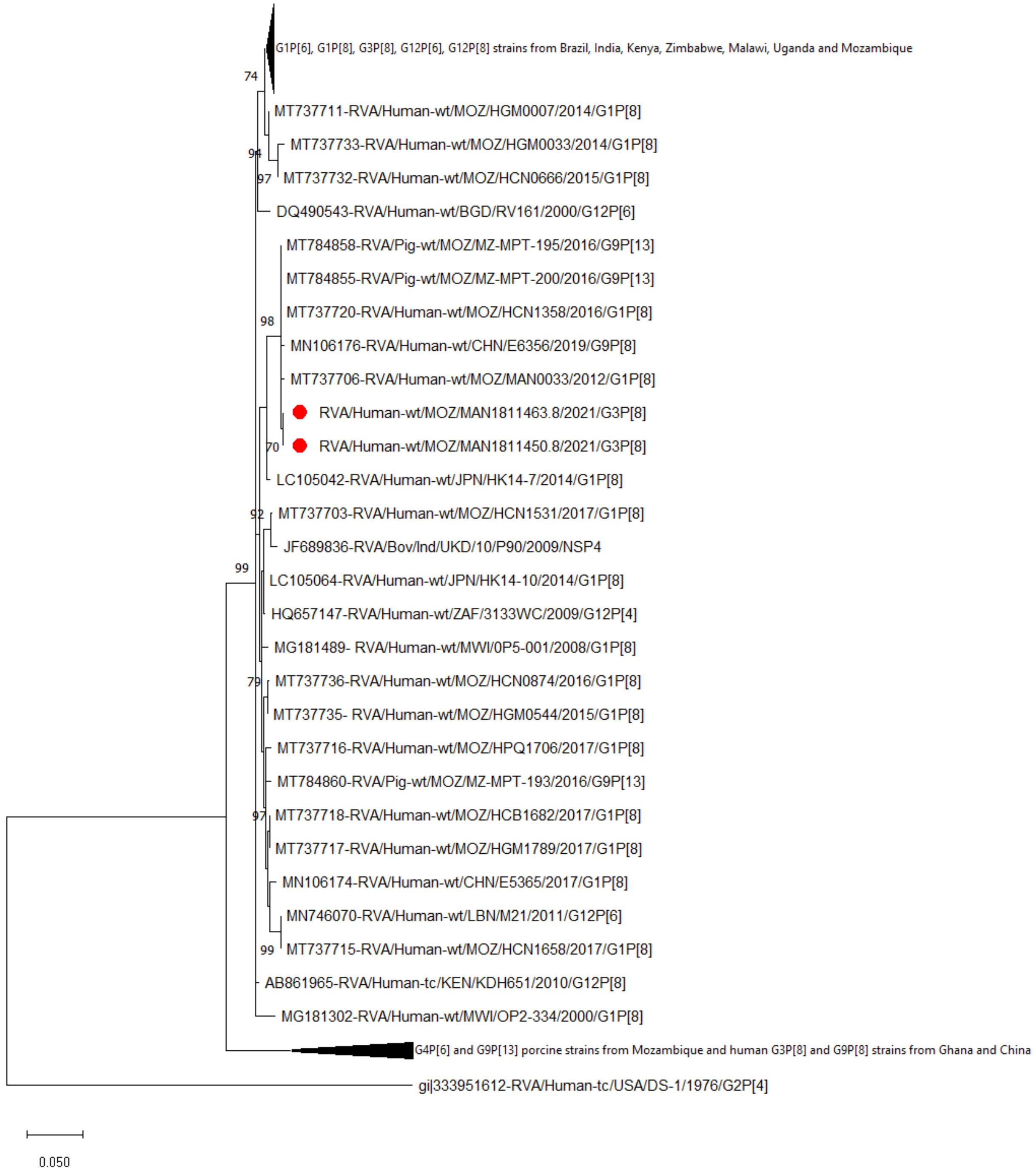

Supplementary Figure H: Phylogenetic tree of NSP4 based on the open reading frame (ORF) nucleotide sequences of RVA/Human-wt/MOZ/MAN-1811463.8/2021/G3P[8] and RVA/Human-wt/MOZ/MAN-1811450.8/2021/G3P[8] strains compared with global strains available from the Genbank. Manhiça strains are indicated by a filled red circle symbol.

I. NSP5/6

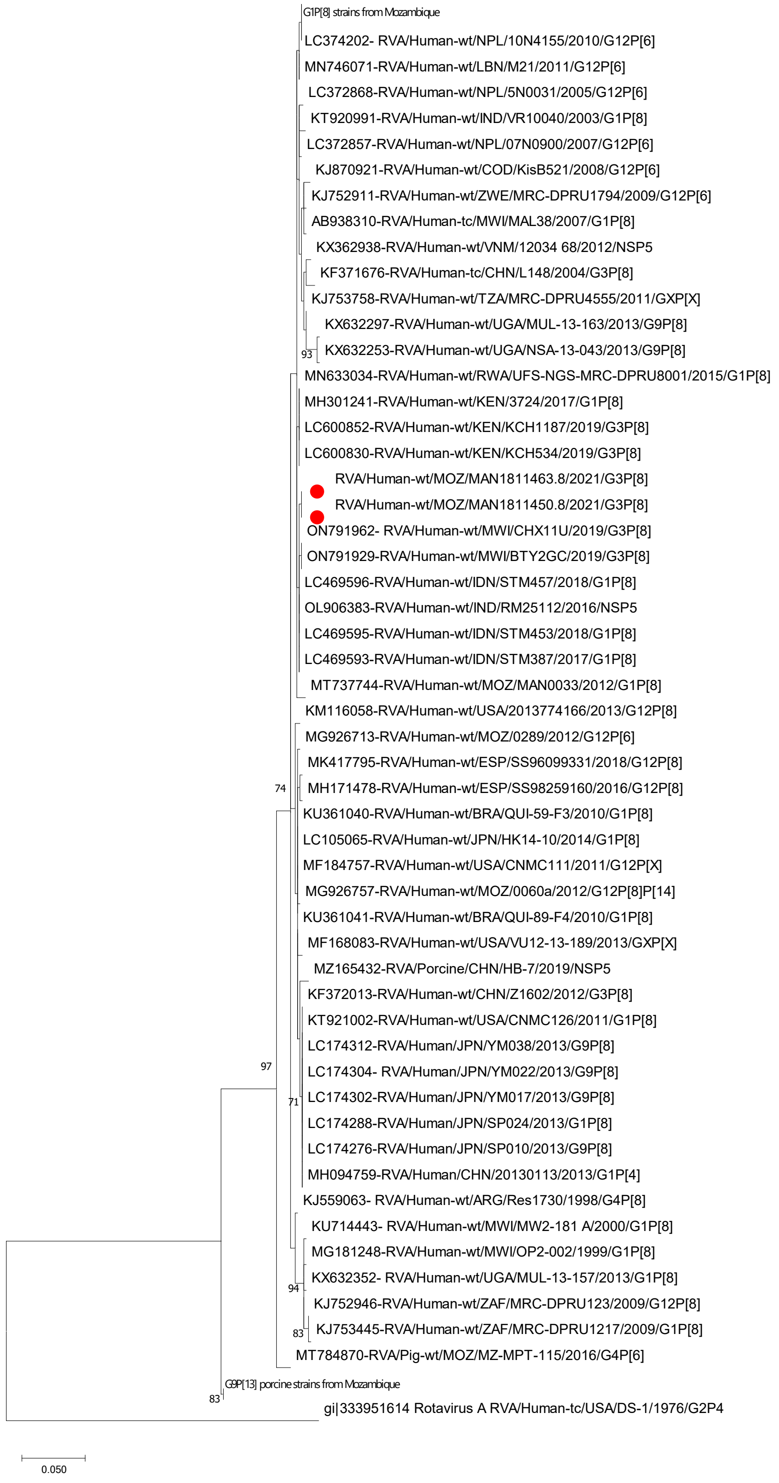

Supplementary Figure H: Phylogenetic tree of NSP4 based on the open reading frame (ORF) nucleotide sequences of RVA/Human-wt/MOZ/MAN-1811463.8/2021/G3P[8] and RVA/Human-wt/MOZ/MAN-1811450.8/2021/G3P[8] strains compared with global strains available from the Genbank. Manhiça strains are indicated by a filled red circle symbol.
